# Clinical Features and Disease Activity in Psoriatic Arthritis: A Sex-Related Perspective on Leptin and Comorbidity

**DOI:** 10.3390/jcm13102959

**Published:** 2024-05-17

**Authors:** Esther Toledano, Luis Gómez-Lechón, Carolina Cristina Chacón, Cristina Hidalgo, Marta Ibáñez, Antonio Márquez, Rubén Queiro, Carlos Montilla

**Affiliations:** 1 Department of Rheumatology, San Carlos Clinical Hospital, 28040 Madrid, Spain; esthertoledano@hotmail.com; 2 Department of Rheumatology, Francesc de Borja Hospital, 46702 Gandía, Spain; lgomezlechon@gmail.com; 3 Department of Rheumatology, Clinical University Hospital of Salamanca, 37007 Salamanca, Spain; caro_chaconv52@hotmail.com (C.C.C.); chidalgoc15@gmail.com (C.H.); martaim2495@gmail.com (M.I.); 4 Department of Physiotherapy, Clinical University Hospital of Salamanca, 37007 Salamanca, Spain; amarvefisio@usal.es; 5 Department of Rheumatology, Hospital Universitario Central de Asturias, 33011 Oviedo, Spain; queiromanuel@uniovi.es

**Keywords:** psoriatic arthritis, sex perspective, comorbidities

## Abstract

**Background/Objectives:** Many studies have addressed the sex differences in patients with psoriatic arthritis, although these are aimed more at describing the phenotype than at investigating the causes underlying these differences. The aims of our study were to assess the presence of clinical features in relation to sex, and to measure the effect on disease activity of different comorbidities in each sex. **Methods:** This was a cross-sectional study in which the following factors were measured: the clinical features of the disease, disease activity, the physical function and the disease impact. We measured serum leptin levels, to eliminate the effect of obesity on leptin levels, and a leptin/BMI ratio was calculated. The comorbid conditions evaluated included anxiety and depression, and sleep quality. **Results:** A total of 203 patients participated in this study. The mean age was 54.6 ± 11.3, and 46.8% of the patients were women. Women less frequently presented axial involvement (8% vs. 28%; *p* < 0.001) and more commonly had enthesitis (2 vs. 0.3; *p* < 0.001). They also had higher DAPSA (16.4 vs. 13.4; *p* < 0.001) and PsAID12 scores (4.1 vs. 2.9; *p* < 0.001), worse HAQ results (0.8 vs. 0.5; *p* < 0.001), and greater FACIT-F scores (32.7 vs. 38.1; *p* < 0.001). As for the comorbid conditions, women presented a higher leptin/BMI ratio (0.8 vs. 0.2; *p* < 0.001), higher levels of HADS-A (6.9 vs. 4.7; *p* < 0.001) and HADS-D (4.9 vs. 3.4; *p* < 0.001), and poorer ISI (9.3 vs. 7.0; *p* < 0.001). By sex, pain affecting women was associated with the leptin/BMI ratio (β: 0.29; *p* < 0.004; 95%CI: 0.3–1.6) and sleep quality (β: 0.31; *p* < 0.004; 95%CI: 0.04–0.25; R^2^: 0.26). The leptin/BMI ratio was not associated with pain in men (*p* = 0.46). **Conclusions:** Sex was associated with several clinical manifestations. Leptin/BMI ratio levels were associated with pain in women, but not in men.

## 1. Introduction

Psoriatic arthritis (PsA) is an immune-mediated musculoskeletal disease that affects both the joints and the entheses. It affects men and women equally, although differences have been found in the phenotype, impact, and response to treatment [1,2]. In fact, multiple joint involvement is more common among women, which is in contrast with men, who are more commonly affected by axial presentations associated with positive HLA-B27 values and greater radiological damage [3,4,5]. Generally, in PsA, HLA-B27 has been found to be associated not only with the susceptibility of the disease with axial involvement, but also with the determination of clinical characteristics, including the earlier onset of psoriasis and arthritis, as well being male, but not with the severity or extent of spondylitis or functional impairment. As for cutaneous manifestations, onychopathy is more frequently reported among men [6]. On the other hand, sex differences in the frequency of comorbidities associated with the disease have been documented. Anxiety, depression, insomnia, or fibromyalgia may affect up to 30% of patients with PsA and, in line with the general population, these comorbid conditions are more frequent among women [4,7,8,9]. Because of their potential effect on pain, these conditions may act as confounding factors for the greater intensity of pain in women. Irrespective of its origin, the intense pain perceived by women leads to greater scores in measures of disease activity (most of which include pain among the domain measure) and to the reduced efficacy and persistence of the treatments administered [10,11,12,13,14]. Despite the potential presence of other pathophysiologic mechanisms that account for the increased perception of pain, most studies continue to accept the presence of these confounders as the only explanation [15]. It is reasonable to think that sex hormones might play a role in these mechanisms. Thus, testosterone, estrogens, and progestogens have been shown to affect pain perception [15,16]. Adipokines are cytokines derived from adipose tissue that play a key role in the generation of metabolic syndromes. These not only contribute to the regulation of insulin-mediated processes, glucose and lipid metabolism, vascular changes, and coagulation; they also participate in chronic inflammation. One of the first adipokines studied in this process is the leptin hormone, which is secreted mainly by adipose tissue, stimulated by ovarian sex hormones and inhibited by testosterone; therefore, the levels of this cytokine in the blood tend to be higher in women [17]. Leptin intervenes in the proinflammatory response by triggering the production of cytokines, such as TNF alpha and IL-6, stimulating differentiation between the Th1 and Th17 cells [18,19,20] and contributing to abnormal pain processing [21]. In terms of metabolic functions, leptin influences glucose metabolism by phosphorylating the insulin receptor and suppressing the glucose transporter which is involved in the development of insulin resistance [22]. In addition, due to its close relationship with lipid metabolism, leptin has been correlated, both in experimental models and in experimental studies, with the presence of non-alcoholic fatty liver disease (NAFLD) [23,24].

In summary, in most studies, the increased disease activity in women with PsA was due to increased pain related to the presence of comorbidities, such as anxiety, depression, or insomnia; however, it is possible that there are sex-related hormonal factors, such as leptin, which through its effect on pain processing may affect disease activity assessment among women with PsA. With this in mind, we analyze the potential role of leptin and other comorbidities in pain perception between sexes.

## 2. Materials and Methods

### 2.1. Type of Study

We performed an observational cross-sectional study at Salamanca University Hospital (Salamanca, Spain).

### 2.2. Population

Inclusion criteria: The study population comprised consecutive patients aged ≥18 years diagnosed with PsA according to the classification criteria for PsA (CASPAR) [25]. All patients were followed up in outpatient rheumatology clinics between March 2023 and November 2023 and agreed to participate in the study.

Exclusion criteria: We excluded the patients who met the American College of Rheumatology diagnostic criteria for fibromyalgia (2016) [26] or who had previously been diagnosed and treated for depression, anxiety, diabetes, or dyslipidemia, in order to rule out the possible influence of these illnesses on the measurements of emotional state and leptin levels [27,28].

This study was approved by the ethics committee of Salamanca University Hospital (EO 2023 03 1246-TFG). The patients gave their written informed consent to participate in the study and for the results derived from the research to be published.

### 2.3. Variables Assessed

#### 2.3.1. Demographic and Clinical Characteristics

Data were collected for the following series of variables: age; sex; years of education; time since onset; smoking status (smoker/former smoker/non-smoker—a former smoker was considered to be a person who had previously been a smoker and had not smoked for at least 12 months), and the number of cigarettes smoked measured in pack-years [29]; treatment with conventional synthetic disease-modifying antirheumatic drugs (csDMARDs), targeted synthetic disease-modifying antirheumatic drugs DMARDs (tsDMARDs), and biologic disease-modifying antirheumatic drugs DMARDs (bDMARDs); the number of patients with > 1 line of treatment with bDMARDs owing to the lack of efficacy; the clinical form of the disease at the time of the study (peripheral, mixed, or axial), with axial presentations defined as inflammatory lower back pain and radiographic damage (sacroiliitis of at least grade 2 as per New York criteria and/or the presence of syndesmophytes) [30,31]; polyarthritis; dactylitis (current or past); and the number of entheses involved as assessed using the modified Maastricht Ankylosing Spondylitis Enthesitis Score (mMASES) [32]. The original MASES [33] takes into account 15 entheses (the bilateral first and seventh costochondral joints, the anterior and posterior superior iliac spine, the iliac crests, the proximal insertion of the Achilles tendons, as well as the fifth lumbar spinous process). The MASES was modified for PsA to include the plantar fascia, with scores ranging from 0 to 15 [32]. The extent of psoriasis was assessed using the Psoriasis Area Severity Index (PASI) [34]. Fatigue was assessed using the Functional Assessment of Chronic Illness Therapy (FACIT) scale, specifically, the FACIT fatigue scale, which has been validated for PsA [35]. Permission to use the instrument was obtained from FACIT.org, accessed on 31 January 2023.

In the case of peripheral involvement, disease activity was measured using the Disease Activity Index for PsA (DAPSA) [36], a composite score designed to assess disease activity in PsA. The DAPSA score is the sum of the C-reactive protein (CRP, mg/dL), tender joint count (0–68), swollen joint count (0–66), the patient global assessment of disease activity (between 0 and 10 on a numerical rating scale [NRS]), and the pain NRS score (0–10). In the case of axial involvement, we used the Ankylosing Spondylitis Disease Activity Score with C-reactive protein (ASDAS-CRP) [37]. We measured functional ability based on the Health Assessment Questionnaire—Disability Index (HAQ-DI) for peripheral involvement and the Bath Ankylosing Spondylitis Functional Index (BASFI) for axial involvement. Disease impact was assessed using the 12-item PsA Impact of Disease questionnaire (PsAID-12) [38,39,40].

#### 2.3.2. Leptin, Emotional State, and Sleep Quality

Leptin levels were determined using an enzyme-linked immunosorbent assay (calibrated according to International Standard WHO/NIBSC 97/594 (recombinant leptin) using a Cobas e411 analyzer with module E170 for modular analytics and Cobas e601 and e602 analyzers). Obesity was measured using the Body Mass Index (BMI), which is a quotient of weight measured in kg and height in squared meters [41]. To eliminate the effect of obesity on leptin levels, the leptin/BMI ratio was performed. Insulin resistance was evaluated through the HOMA (homeostatic model assessment) index, using the following formula: fasting plasma glucose (mg/dL) × fasting plasma insulin (IU/mL)/405 [42]. The HOMA index yields an estimate of insulin sensitivity and β-cell function and represents a good indicator for insulin resistance to be used in epidemiological studies. The higher the value, the higher the severity of insulin resistance, with cut-offs variably defined between 1.8 and 3.8 indicating a pathologically altered insulin sensitivity [43]. The presence of NAFLD was obtained using the fibrosis-4 index. The FIB-4 index was calculated to estimate liver fibrosis. This index includes age, transaminases and platelets: a score < 1.3 points rules out advanced fibrosis (F0–F1), a score > 2.67 points implies significant fibrosis (F3–F4), and a score between 1.3 and 2.67 points is an intermediate zone (F2) in which another diagnostic test must be performed. The FIB-4 score was categorized as a dichotomous variable into normal (F0-1, non-advanced fibrosis zone) and altered (F2-3-4, intermediate and significant fibrosis zones) for analysis purposes [44].

Emotional factors were assessed using the Hospital Anxiety and Depression Scale (HADS), a 14-item scale applied to determine which individuals among those with medical conditions had anxiety and depression. Scores range from 0 to 21 for each subscale (HADS-D for depression and HADS-A for anxiety) and are classified as normal (0–7), borderline abnormal indicating a possible clinical disorder (8–10), and abnormal indicating a probable clinical disorder (11–21) [45].

The Insomnia Severity Index (ISI) was applied to assess sleep quality. ISI is a self-administered questionnaire that includes 7 items that address the nature, severity, and impact of insomnia. Responses are on a 5-point Likert-type scale (0 to 4) for the previous month. The overall score ranges between 0 and 28 and is classified as no clinically significant insomnia (0–7), subthreshold insomnia (8–14), clinical insomnia (moderate severity) (15–21), and clinical insomnia (severe) (22–28) [46].

#### 2.3.3. Statistical Analysis

The quantitative variables are reported as the mean and standard deviation, and the categorical variables are shown as numbers and percentages. The groups were compared using the t-test for normally distributed quantitative variables and the Mann–Whitney test for the ordinal variables or non-normally distributed quantitative variables. Comparisons between more than two groups were performed using one-factor analysis of variance (normally distributed quantitative variables) and the Kruskal–Wallis test (ordinal variables or non-normally distributed quantitative variables). The normally distributed variables were summarized using the mean ± standard deviation (SD), and the non-normally distributed variables were represented by the median and interquartile range (IQR). Pearson’s correlation coefficient was applied to determine the correlations between quantitative variables. The statistical significance was set at *p* < 0.05.

We examined the bivariate correlations between the leptin/BMI ratio, ISI, HADS-A, HADS-D, and components of DAPSA for both sexes. As leptin exerts an effect on pain [21], we applied linear regression analysis for both the sexes, with the dependent variable being pain according to the visual analog scale (VAS), and the independent variables being the leptin/BMI ratio and the ISI, HADS-A, and HADS-D scores. Both the models were adjusted for treatment with tsDMARDs and bDMARDs. The independent variables were selected as per previous findings [9,47].

This analysis was performed using IBM SPSS Statistics for Windows, Version 23.0.

## 3. Results

### 3.1. Demographic and Clinical Characteristics

The mean age was 54.6 ± 11.3 years, and 46.8% of the patients were women. The time since the onset of the disease was 10.0 ± 7.0 years, and 27.1% of the patients were receiving tsDMARDS or bDMARDs. The mean DAPSA score was 14.9 ± 7.4. The remaining data are summarized in Table 1.

### 3.2. Demographic and Clinical Variables, Disease Activity, Functioning, Disease Impact, Leptin Levels and Comorbid Conditions: Comparison between the Sexes

Axial involvement was less common and enthesitis was more common among the women. The women also had more marked disease activity and impacts and poorer functioning. No differences in BMI were found between the sexes. The women had a greater leptin/BMI ratio and more severe fatigue. As for the remaining comorbid conditions, the women had higher degrees of anxiety, depression, and fatigue and poorer sleep quality.

The results of the comparisons are summarized in Table 1.

#### Association between Comorbid Conditions and Disease Activity by Sex

- Women

The leptin/BMI ratio (r: 0.2 *p* < 0.02), and the HADS-D (r: 0.2 *p* < 0.005) and ISI (r: 0.3 *p* < 0.001) scores all correlated with VAS pain. The rest of the results are presented in Table 2.

Linear regression analysis showed pain to be associated with the leptin/BMI ratio (β: 0.29; *p* < 0.004; 95%CI: 0.3–1.6) and sleep quality (β: 0.31; *p* < 0.004; 95%CI: 0.04–0.25; R^2^: 0.26). The values for HADS-A and HADS-D were *p* = 0.4 and *p* = 0.09, respectively.

- Men

The leptin/BMI ratio was not correlated with VAS pain (r: 0.07 *p* = 0.46). The rest of the results are presented in Table 3.

Linear regression analysis showed that pain was associated with sleep quality (ISI β: 0.4; *p* < 0.001; 95%CI: 0.15–0.41; R^2^: 0.27). The values for the remaining variables were as follows: leptin/BMI (*p* = 0.7), HADS-A (*p* = 0.9), and HADS-D (*p* = 0.4).

Missing data accounted for less than 3%.

## 4. Discussion

Many studies have addressed the sex differences in patients with PsA; however, research has focused more on describing the phenotype than investigating the causes underlying sex differences. We found sex-related differences regarding disease activity and functioning, but these differences were mostly explained by subjective variables, such as pain. Higher leptin concentrations, while not directly associated with obesity, may have a role in the perception of pain among women.

In most previous studies, clinical presentations differed between the sexes, mainly in the greater frequency of axial disease affecting men [3,5]. Consistent with previous reports, we found a greater presence of axial disease, defined according to the degree of radiological damage. Furthermore, Eder et al. [3] found being male was associated not only with more frequent axial presentations, but also with greater radiological damage. However, these results were not confirmed in the recent studies on Turkish and Chinese populations [48,49]. In contrast with the data reported by Queiro et al. [5], our results are similar to those from various cohort studies [3,48,49], which did not reveal more frequent polyarticular presentations among women. As for the other clinical manifestations, enthesitis was more common among women. These results are consistent with those from the cohorts CORRONA [50] and ASAS [51]. However, most studies reported no association between sex and the predominance of enthesitis. These differences can be explained by the various methods of measurement applied [48,49]. As in the other studies, we found no association between sex differences and dactylitis or the severity of psoriasis [3,4,5,48,49].

The disease impact was also greater among the women, which is consistent with the data reported by Gossec et al. [4].

As for comorbidity, we did not find statistically significant differences in BMI between the sexes. Some previous studies report greater BMI among women [48,51], possibly more so because of the inclusion criteria than the presence of any real sex differences.

Due to the higher levels of leptin among women and its demonstrated link to pain in animal models [21,52], we found it interesting to include this determination in order to measure its sex-related influence on the disease activity parameters. We found that the leptin levels in women, adjusted for BMI, were associated with pain intensity. Although studies have researched the effect of this hormone on joint manifestations in patients with PsA, to our knowledge, these sex-related differences have not been studied to date. Eder et al. [3] found no differences in leptin levels and the number of active joints, although there was a slight trend in this relationship (r:0.10; *p* = 0.05). In another study, these authors found no association between pain intensity and the leptin levels [53]. A recent study by our group found higher serum leptin levels in the patients with neuropathic pain, as determined by the Pain-DETECT questionnaire (26.8 vs. 14.2; *p* < 0.001) [54]. In other diseases, a recent study of patients with rheumatoid arthritis found an association between leptin levels and pain, although the authors did not analyze the results by sex [55]. This association has also been found in patients with osteoarthritis [56]. However, leptin has been shown to have pro-inflammatory properties, being associated with the increased secretion of several interleukins. In our study, we found no correlation in either sex between the leptin levels and inflammation-related components.

As for the axial manifestations, Hernández-Breijo et al. [57] studied a cohort of patients with axial spondyloarthritis and reported an association between the response to treatment with TNF inhibitors and baseline leptin levels. We found no correlation between axial disease activity and the leptin/BMI ratio. This observation could be explained by the small number of patients included in this subgroup.

On the other hand, in our study we found no difference between the sexes regarding glucose resistance. There are not many articles that analyze the sex perspective related to the presence of diabetes mellitus (DM) in patients with PsA. Dreiher et al. analyzed the difference in risk of DM between male and female patients affected by PsA. Females in the PsA group had a DM prevalence of 18.7% (10.3% of the control group) with OR 1.60, while in males there was the same prevalence (11.2%) in the PsA group and in the control group with OR 0.71 [58]. Although we excluded patients with DM, we found a correlation between leptin levels and HOMA index scores in both women (r = 0.4; *p* < 0.001) and men (r = 0.3; *p* = 0.005). It has also been shown that increased levels of serum leptin could be used as a risk factor in the development of type 2 diabetes mellitus [59].

The prevalence of NAFLD in PsA is higher than that found in other chronic joint diseases [60]. Its pathogenesis is influenced by the interplay of several factors including leptin [61,62]. In our study, we found that 28.6% of patients had some degree of liver fibrosis, results similar to those recently published by Ortolan et al. [63]. We found no difference between the sexes. These results are consistent with those previously published [63,64]. In our study, patients with liver fibrosis did not have higher leptin levels (14.2 vs. 11.2; *p* = 0.2). These results may be due to the fact that we did not exclude other causes of liver impairment such as methotrexate use or alcoholism.

Although we excluded patients with fibromyalgia, anxiety, and depression, the scores on the questionnaires associated with these conditions, either directly or indirectly, were higher among the women. Moreover, they affected various items on the DAPSA in both sexes. Anxiety and depression have been associated with increased disease activity, especially in terms of the subjective components of the instrument [47]. However, they more frequently affect women, irrespective of whether they have PsA, meaning that they must be considered confounders and not a sex-associated characteristic of the disease. Furthermore, these comorbid conditions affected the subjective variables of disease activity for both the sexes, although they were more frequent among the women. As for sleep quality, while this can be considered to be closely linked to anxiety and depression, a recent study by our group found an association between sleep quality and disease activity, irrespective of these conditions [9]. To our knowledge, no other studies have previously associated sleep quality with sex in PsA.

Our study is limited by its cross-sectional design, and although it is not possible to establish causal relationships, in the case of the association between the leptin/BMI ratio and intensity of pain, the association favors the effect of leptin on intensity, and not the reverse. Furthermore, although in regression analysis it was more influential than the other comorbidities, it should be remembered that the correlation with pain was weak. Studies with other characteristics could better consolidate the effect of higher leptin concentrations in women with more pronounced disease activity, without considering their role in obesity. Our study was also limited by the fact that we did not investigate the women’s hormonal status, even though the secretion of leptin is known to be associated with estrogen production. Lastly, while there is a direct correlation between BMI and leptin secretion, BMI may not be the best marker to accurately reflect adiposity leading to greater leptin secretion. Future studies will include diabetes, given the association of this comorbidity with psoriatic arthritis and metabolic syndrome.

Despite its limitations, to our knowledge our study is the first to address the role of leptin, a hormone whose secretion is closely linked to sex, as a possible cause of the difference in disease activity observed in women with PsA.

## 5. Conclusions

In line with previous research, our work showed that sex plays a significant role in the subjective parameters associated with activity, functionality, and disease impact. In contrast, from a novel perspective, we found that higher leptin concentrations—a characteristic of being female and not of obesity—could influence pain perception among women. Specially designed longitudinal studies, including sequential measurements of both the parameters and monitoring their progression over time, could clarify whether leptin, a hormone whose secretion predominates among females and that has been shown in other contexts to be related to pain, may play a role in the increased sensitivity to pain in our female patients. If confirmed, this finding could help to explain the sex differences in pain mechanisms and serve as a basis for further research into whether this hormone could be considered as a prognostic factor related to the presence of pain.

## Figures and Tables

**Table 1:** Demographic, clinical, and disease-related characteristics of patients with PsA by sex.

Variable	All (*n* = 203)	Women (*n* = 95)	Men (*n* = 108)	*p*
Age *	54.6 ± 11.3	54.2 ± 10.2	55.0 ± 12.3	0.42
Years of education **	13.0 (9)	10.0 (8)	10 (10)	0.53
Years since onset **	14.1 (18)	7.3 (8)	15 (30)	0.27
Smoking status *n* (%)				
Smoker	53 (26)	33 (35)	20 (18)	0.001
Former smoker	93 (46)	31 (33)	62 (57)	0.001
Non-smoker	57 (28)	31 (32)	26 (24)	0.01
Smoking, pack-years	12.9 (20)	12.9 (20)	15 (30.1)	0.13
Conventional synthetic DMARDs	153 (75)	78 (82)	75 (79)	0.15
Methotrexate	105 (52)	51 (54)	54 (50)	
Sulfasalazine	38 (19)	22 (23)	16 (15)	
Leflunomide	10 (5)	5 (5)	5 (5)	
Apremilast	4 (2)	2 (2)	2 (2)	
tsDMARDs or bDMARDs, *n* (%)	55 (29)	25 (25)	30 (28)	0.53
TNF inhibitor	34 (17)	13 (14)	21 (19)	
Secukinumab	14 (7)	8 (8)	6 (6)	
Ustekinumab	3 (1)	1 (1)	2 (2)	
Tofacitinib	4 (2)	3 (3)	1 (1)	
Failure of tsDMARDs or bDMARDs, *n* (%)	28 (50)	16 (64)	12 (38)	0.06
Clinical presentation, *n* (%)				0.001
Peripheral	166 (82)	88 (92)	78 (72)	
Mixed	31 (15)	7 (8)	24 (22)	
Axial	6 (3)	0 (0)	6 (6)	
Polyarthritis (yes/no) (%)	20/183 (9.9)	8/87 (8)	12/96 (11)	0.46
Dactylitis (yes/no) (%)	36/167 (18)	12/83 (12)	24/84 (22)	0.07
mMASES **	0.5 (1.2)	2 (3.7)	0.3 (1)	0.001
PASI **	0.6 (1.2)	0.8 (0.5)	1 (1.8)	0.23
FACIT-F *	35.8 + 11.3	32.7 ± 11.2	38.5 ± 10.8	0.001
CRP (mg/dL) **	1.1 (1.7)	0.3 (1.4)	0.4 (1)	0.45
Pain VAS **.	5.5 (4)	6 (3)	4 (5)	0.001
Activity VAS **	5.5 (4.5)	5 (4)	3 (5)	0.03
SJC **	1.5 (4)	1 (2.7)	1 (2)	0.72
TJC **	3.5 (4)	4.5 (3)	3 (3.5)	0.02
DAPSA *	14.9 ± 7.4	16.4 ± 7.1	13.4 ± 7.5	0.001
ASDAS-CRP *	1.7 ± 0.8	2.4 ± 0.8	1.5 ± 0.7	0.02
HAQ-DI *	0.6 ± 0.6	0.8 ± 0. 5	0.5 ± 0.5	0.001
BASFI *	3.5 ± 2.8	4.9 ± 2.5	3.0 ± 2.8	0.95
PsAID-12 **	4.2 (4.3)	4.1 (3.2)	2.9 (2.6)	0.001
BMI (kg/m^2^) *	27.0 ± 4.4	26.7 ± 5.2	27.3 ± 3.5	0.06
Leptin (ng/mL) **	10.7 (11.8)	20.6 (31)	5.6 (11.3)	0.001
Leptin/BMI **	0.3 (0.3)	0.8 (0.8)	0.2 (0.3)	0.001
HOMA	1.9(1.7)	1.7(1.7)	2.1(1.7)	0.25
FIB-4 (normal/altered)	145/58	73/22	72/36	0.1
HAS-A *	5.7 ± 3.7	6.9 ± 3.8	4.7 ± 3.2	0.001
HAS-D *	4.1 ± 3.6	4.9 ± 3.4	3.4 ± 3.5	0.004
ISI *	8.1 ± 4.7	9.3 ± 4.9	7.0 ± 4.3	0.001

Abbreviations: CRP: C-reactive protein; ASDAS-CRP: Ankylosing Spondylitis Disease Activity Score with C-reactive protein; BASFI: Bath Ankylosing Spondylitis Functional Index; DAPSA: Disease Activity Index for PsA; tsDMARD: targeted synthetic disease-modifying antirheumatic drug; bDMARD: biologic disease-modifying antirheumatic drug; FACIT: Functional Assessment of Chronic Illness Therapy; HADS: Hospital Anxiety and Depression Scale; HAQ-DI: Health Assessment Questionnaire-Disability Index; ISI: Insomnia Severity Index; SJC: swollen joint count; TNF: tumor necrosis factor; TJC: tender joint count; VAS: visual analog scale; BMI: body mass index; HOMA: homeostatic model assessment; FIB-4: fibrosis-4 index; mMASES: modified Maastricht Ankylosing Spondylitis Enthesitis Score; PASI: Psoriasis Area Severity Index; PsAID-12: 12-item PsA Impact of Disease questionnaire. * Mean/SD. ** Median/IQR.

**Table 2:** Correlation between components of DAPSA and leptin/BMI levels, HADS, and ISI in women.

	CRP	VAS Pain	VAS Activity	TJC	SJC
Leptin/BMI	r: 0.0 *p* = 0.81	r: 0.2 *p* = 0.02	r: 0.1 *p* = 0.13	r: 0.0 *p* = 0.55	r: 0.1 *p* = 0.12
HADS-A	r: 0.0 *p* = 0.82	r: 0.1 *p* = 0.09	r:0.2 *p* = 0.03	r: 0.0 *p* = 0.96	r: 0.0 *p* = 0.92
HADS-D	r: 0.1 *p* = 0.13	r: 0.2 *p* = 0.005	r: 0.2 *p* = 0.02	r: 0.1 *p* = 0.08	r: 0.2 *p* = 0.04
ISI	r: 0.0 *p* = 0.92	r: 0.3 *p* < 0.001	r: 0.1 *p* = 0.14	r: 0.2 *p* = 0.01	r: 0.2 *p* = 0.04

Abbreviations: BMI: body mass index; CRP: C-reactive protein; HADS: Hospital Anxiety and Depression Scale; ISI: Insomnia Severity Index; SJC: swollen joint count; TJC: tender joint count, VAS: visual analog scale.

**Table 3:** Correlation between components of DAPSA and leptin/BMI levels, HADS, and ISI in men.

	CRP	VAS Pain	VAS Activity	TJC	SJC
Leptin/BMI	r: 0.02 *p* = 0.71	r: 0.07 *p* = 0.46	r: 0.0 *p* = 0.47	r: 0.02 *p* = 0.75	r: 0.06 *p* = 0.52
HADS-A	r: −0.0 *p* = 0.33	r: 0.37 *p* < 0.001	r: 0.1 *p* = 0.22	r: 0.0 *p* = 0.33	r: 0.0 *p* = 0.72
HADS-D	r: −0.1 *p* = 0.24	r: 0.4 *p* = 0.005	r: 0.1 *p* = 0.06	r: 0.0 *p* = 0.35	r: −0.1p = 0.84
ISI	r: -0.7 *p* = 0.45	r: 0.51 *p* < 0.001	r: 0.36 *p* < 0.001	r: 0.30 *p* = 0.01	r: −0.0 *p* = 0.55

Abbreviations: BMI: body mass index; CRP: C-reactive protein; HADS: Hospital Anxiety and Depression Scale; ISI: Insomnia Severity Index; SJC: swollen joint count; TJC: tender joint count, VAS: visual analog scale.

## Data Availability

The data presented in this study are available on request from the corresponding author.
